# Association between Poor Quality of Sleep and Metabolic Syndrome in Ghanaian University Students: A Cross-Sectional Study

**DOI:** 10.1155/2022/8802757

**Published:** 2022-10-12

**Authors:** Kwame Yeboah, Kennedy K. Dodam, Jennifer A. Agyekum, Jared N. Oblitey

**Affiliations:** ^1^Department of Physiology, University of Ghana Medical School, P O Box, 4236 Accra, Ghana; ^2^Department of Adult Health, School of Nursing, University of Ghana, P O Box LG 25, Legon, Accra, Ghana; ^3^Medical Laboratory Unit, Mamprobi Hospital, Accra, Ghana; ^4^Department of Radiography, School of Biomedical and Allied Health Sciences, University of Ghana, P O Box KB139, Accra, Ghana

## Abstract

**Aim:**

This study aimed to determine the association between quality of sleep and metabolic syndrome (MetS) and physical activity level in young adults at the University of Ghana.

**Method:**

In a cross-sectional design, 340 university students, aged between 20-30 years were recruited. Quality of sleep was assessed using the Pittsburgh Sleep Quality index (PSQI) and physical activity with the short form of the International Physical Activity Questionnaire (IPAQ-SF). Poor quality of sleep was defined as a global PSQI score>5 and low physical activity level as those not meeting the criteria for vigorous-moderate physical activity. Anthropometric features and blood pressures were measured, and fasting blood samples were collected from the participants to measure plasma levels of glucose, lipid profile, urea, and creatinine. MetS was defined using the Joint Scientific Statement criteria.

**Results:**

In our study population of young adults from Ghana, the prevalence of poor quality of sleep as measured by PSQI was 54.1%, and MetS was 12.4%. MetS was associated with poor quality of sleep in females [OR (95%CI) = 2.11 (1.04–4.25), *p* = 0.038] and entire study participants [2.18 (1.09–4.37) *p* = 0.029] in both crude and adjusted models; no association was found in male participants. Low physical activity status was not associated with poor sleep status. Obesity [1.32 (1.02–3.56), *p* = 0.043], but not overweight [0.99 (0.58–2.34), *p* = 0.862], was associated with poor quality of sleep.

**Conclusion:**

Young adults in a Ghanaian university have a high prevalence of poor quality of sleep and is associated with MetS and obesity. Physical activity status was not associated with poor quality of sleep.

## 1. Introduction

Metabolic syndrome (MetS) is the clustering of metabolic abnormalities such as dyslipidaemia, abdominal obesity, elevated plasma glucose, and blood pressure [1–3]. A meta-analysis reported that the prevalence of MetS in the healthy Ghanaian population based on the National Cholesterol Education Program, Adult Treatment Panel, World Health Organization, or International Diabetes Federation classifications were 12.4% 6.0% and 21.2%, respectively [1]. A similar prevalence of MetS has been reported in a recent meta-analysis of the sub-Saharan African population [4]. MetS has been linked to an increased risk for developing cardiovascular diseases (CVDs) and type 2 diabetes [3, 5]. The underlying aetiology of MetS is multifactorial but not well known [2], with prominent contributions from sedentary lifestyles [6–8] and psychological factors [9] including depression and quality of sleep [10–12]. Given the high prevalence and serious complications of MetS, early identifying and controlling the modified risk factors are crucial prevention methods against the development of MetS and its progression to CVDs.

Sleep health affects human physiological mechanisms and may contribute to overall health [6, 13]. Sleep health is normally reported in two forms as duration and quality, with both forms associated with MetS, CVDs, and mental illness [14–17]. Poor quality of sleep alters sympathovagal regulation, leading to an exaggeration of sympathetic nervous system activity [13, 14] which may inhibit insulin secretion, promote insulin resistance, and contribute to the development of MetS [2, 5]. Sleep disturbances have traditionally been considered a byproduct of mental health disorders. Current evidence also proposes that sleep problems or difficulties can contribute to the development of different mental health problems as well as to the persistence of those already present. Moderate-vigorous physical activity has been shown to improve sleep health and overall quality of life in patients and healthy population [7, 14, 15].

University years represent a duration of transitioning from adolescence to adulthood in which students have reduced parental support and increased stress from academic loads and lifestyle, leading to increased anxiety, sleep problems, and reduced mental health. In Ghana, many individuals may first be exposed to “westernized” lifestyles such as poor eating habits, increased usage of social media on electronic devices, and physical inactivity, which can prone them to poor sleep health and MetS during the university years. We hypothesized that poor quality of sleep and low physical activity level may be associated with MetS in university students in Ghana.

## 2. Methods

### 2.1. Study Design

In a cross-sectional design study conducted from November 2018 through May 2019, we recruited male and female students within the age range of 20–30 years from the University of Ghana, Accra. Potential participants were invited electronically through SherlockMD online application (http://www.sherlockmd.org). Participants with already diagnosed diabetes, cardiopulmonary diseases, prior vascular surgery, and those who could not come to the study centre to provide voluntary informed consent form were excluded from the study. In all, 408 out of 556 students (73.4% respondent rate) responded favourably to our invitation by filling the online questionnaire. Out of these, 370 students visited the study centre to provide written voluntary informed consent, and for measurement of their blood pressure, anthropometry, as well as blood sample collection. During analysis, 30 questionnaires were excluded because they were incomplete or wrongly filled. In final analysis, data from 340 students was used. The study was conducted in conformity with the Helsinki Declaration on Human Experimentation, 1964 with subsequent revisions, latest Seoul, October 2008. Institutional approval was granted by the Ethical and Protocol Review Committee of the College of Health Sciences, University of Ghana (MS-ET/M.3-P.3.3/2016-2017).

### 2.2. Assessment of Lifestyle, Quality of Sleep, and Physical Activity

After informed consent, a structured questionnaire was used to collect socio-demographic and lifestyle data (age, gender, personal medical history, smoking, and alcohol intake). Sleep quality was assessed using the Pittsburgh Sleep Quality index (PSQI), which is a nineteen-item, validated questionnaire that measures self-reported sleep quality over the past month. This questionnaire has been used in studies involving college students from other countries [11, 18–21]. The PSQI questions were combined into seven different scores ranging from 0 (no difficulty) to 3 (severe difficulty) on the topics of sleep quality, sleep latency, sleep duration, sleep efficiency, sleep disturbances, sleep medication, and daytime dysfunction per the PSQI scoring guidelines. The seven component scores were summed for a final PSQI score that ranged from 0 to 21, with sleep quality declining with each increase in the score. PSQI score>5 is indicative of poor sleep quality.

The level of physical activity in the study participants International Physical Activity Questionnaire-short form (IPAQ-SF) was used to assess the. The IPAQ-SF captured the number of days and time spent on physical activity in vigorous-intensity, moderate-intensity, and walking of at least 10 min duration over the last 7 days, and includes time spent sitting over the last 7 weekdays [22]. The IPAQ-SF sum score is expressed in Metabolic Equivalent of Task (MET)-minutes per day or week. Participants were categorized as those fulfilling or not fulfilling the physical activity (PA) recommendations, moderate-vigorous PA (MVPA)-minutes (≥150 or<150 MVPA-minutes per week), and vigorous-intensity PA (≥75 or<75 min per week) were dichotomized.

### 2.3. Anthropometry and Blood Pressure

Weight, height, waist, and hip circumferences were measured using a standard protocol. Body mass index (BMI) was computed as weight (kg) divided by height squared (m^2^). Percentage body fat and visceral fat were estimated through bioelectrical impedance analysis with the Body composition monitor (BF-508, Omron Healthcare, Inc., Vernon Hills, IL, USA). Blood pressure (BP) was measured after 5 min rest, with the participant in a seated position with back support, by using an automated BP monitor (HEM-705CP; Omron Corporation, Tokyo, Japan). Three different measurements were taken and the last two averaged for analysis.

### 2.4. Biochemical Analysis

After 8–12 h of overnight fasting, approximately 10 ml of venous blood was drawn from the antecubital fossa into appropriate tubes. The samples were centrifuged and serum/plasma aliquoted for analysis. Fasting plasma glucose (FPG), total cholesterol, high-density (HDL) lipoprotein cholesterol, triglycerides, plasma urea, and serum creatinine levels were analysed using a chemical autoanalyser (Mindray BS 200, China), and commercial reagents (Randox Laboratory Reagents, UK). Low-density lipoprotein (LDL) cholesterol levels were calculated using Friedewald's formula.

### 2.5. Definition of Metabolic Syndrome

MetS was defined, according to the Joint Scientific Statement criteria, as individuals with any three or more of the following five components: (1) abdominal obesity (waist circumference ≥94 cm for men and ≥80 cm for women); (2) high triglycerides ≥1.7 mmol/L; (3) low HDL cholesterol: men <1.0 mmol/L or women <1.3 mmol/L; and (4) High BP (systolic BP ≥130 mmHg and/or diastolic BP ≥85 mmHg); and (5) impaired fasting glucose ≥5.6 mmol/l.

### 2.6. Statistical Analysis

Continuous variables that are normally distributed such as age, anthropometric indices, blood pressures, and biochemical parameters were analysed using an unpaired *t*-test and those nonnormally distributed such as levels of physical activity were analysed using Mann-Witney *U* test. Chi-square test was used to analyse drinking and smoking habits, as well as the prevalence of poor quality of sleep, physical activity, MetS, and its components such as abdominal obesity, hypertension, reduced HDL cholesterol, impaired glucose tolerance, and dyslipidaemia. Logistic regression analyses were performed to assess the association between poor quality of sleep with MetS, physical activity level, and obesity after adjustment for plausible confounding factors like age, gender, smoking, and alcohol status. Odds ratios were calculated from logistic regression analysis with 95% confidence intervals. All analyses were performed using SPSS version 27, and *p* < 0.05 was considered statistically significant.

## 3. Results

**Figure 1:**
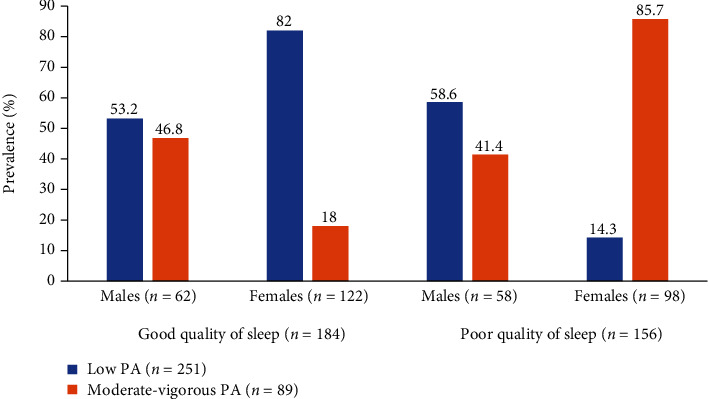
Prevalence of physical activity (PA) status by gender and quality of sleep in study participants.

**Figure 2:**
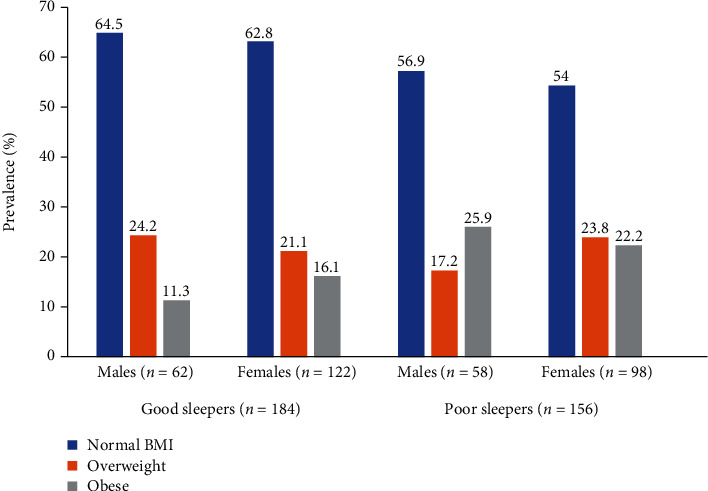
Prevalence of obesity across various gender by sleep status.

The prevalence of MetS was higher in participants with poor quality of sleep compared to participants with good quality of sleep. Participants with poor quality of sleep had higher mean weight, BMI, waist-hip ratio, and mean BP compared to those with good quality of sleep. For the lipid profile, participants with poor quality of sleep had higher levels of total and reduced HDL cholesterol compared to those with good quality of sleep (Table 1). When physical activity levels were categorised into low versus moderate-vigorous, there were high proportions of female participants (73.3% vs 40.4%, *p* < 0.001) with low physical activity levels compared to their male counterparts (26.7% vs 59.6%, *p* < 0.001), respectively. Physical activity status was not associated with quality of sleep in male and female participants (Figure 1). BMI categorisation was not associated with sleep status in both male and female participants (Figure 2).

The prevalence of MetS in our study participants was 12.4%, higher in females than males. When the components of sleep quality were compared based on MetS status, MetS participants had lower subjective sleep quality and higher sleep habituation efficiency scores and sleep medication and global scores (Table 2). When components of MetS were compared based on the quality of sleep, high BP, abdominal obesity, and reduced HDL cholesterol were associated with increased odds of poor quality of sleep in the entire study participants. Abdominal obesity and reduced HDL cholesterol were associated with increased odds in female participants. There was no association between any of the components of MetS and sleep status in male participants (Table 3).

Finally, in a multivariable logistic regression model, the presence of MetS was associated with poor quality of sleep in females and entire study participants in both crude and adjusted models; no association was found in male participants. Low physical activity status was not associated with poor sleep status. Concerning BMI categorisation, overweight and obese male and female participants had no significant increase in odds ratio when compared to participants with normal BMI. When all participants were pooled together, obese participants, but not overweight participants, had an increase in odds when compared to normal BMI participants in both crude and adjusted models (Table 4).

## 4. Discussion

From our literature search, this is the first study to report the relationship between MetS and self-reported quality of sleep in the sub-Saharan Africa population. In our study population of young adults from Ghana, the prevalence of poor quality of sleep as measured by PQSI was 54.1%, and MetS was 12.4%. However, the level of physical activity measured by IPAQ was not associated with the quality of sleep. Obesity, but not overweight, was associated with poor quality of sleep. We have previously reported and discussed MetS in this study population [23]. The prevalence of self-reported poor quality of sleep in our study is similar to the 50.6% and 55.8% reported in University students in Nigeria [20] and Ethiopia [12], respectively, but lower than the 95.3%, 76%, 65.9%, and 59.4% reported in university students in the Fortaleza, Brazil [12], medical students in Saudi Arabia [24], Lithuanian university students [25] and American college students [11], respectively. All these studies used similar instruments and cut-offs to assess poor sleep quality as in our study. The variation in the prevalence of poor sleep quality may be attributed to the socio-cultural differences that affect sleep in different geographical places.

The findings of this study also indicate that the presence of MetS increased the odds of poor quality of sleep in the entire study population and female participants, but not in male participants. Most studies from our literature search have reported on the association between MetS and sleep focused on children, adolescents [26, 27], patients, or adult populations [16, 17]. All these studies and meta-analysis reported an association between the presence of MetS and poor quality of sleep. Consistent with the findings of this study, Araújo et al. (2015) reported that among university students in Brazil, poor quality of sleep was associated with a 5% increase in odds of having MetS [12]. The mechanisms underlying MetS and poor quality of sleep are unclear, but it is known that insufficient or poor quality of sleep can disrupt homeostatic functioning, resulting in increased food intake by raising appetite through dysregulation of hormones that increase hunger and decrease satiety, and lead to lower physical activity due to fatigue [10, 16]. Poor sleep quality can also affect glucose homeostasis, and experimental evidence has shown that sleep restriction can induce insulin resistance likely through inflammatory pathways and epigenetic changes to the expression of circadian clock genes that regulate biological processes [14, 28]. Furthermore, sleep restriction raises blood pressure through multiple potential mechanisms like higher catecholamine production and hyperactivity of the sympathetic nervous system [14, 24, 29]. Nonetheless, objective measures of sleep and MetS coupled with biomarkers of physiological functioning are necessary to confirm these mechanistic pathways.

In this study, participants with MetS had lower subjective sleep efficiency and higher sleep problems as indicated by higher scores for habitual sleep efficiency, sleep medication usage, and global PQSI score. Other studies reported sleep problems that included some of the sleep problems identified in our study. In the Qazvin Metabolic Disease Study conducted in Iran, participants with MetS had higher scores for sleep duration, sleep disturbances, and sleep medication usage than those without MetS [10]. In the Japanese population, it was reported that, compared to those without MetS, males with MetS had higher global PSQI score, sleep latency score, sleep duration score, and sleep disturbance score, while females with MetS also had higher global PSQI score, sleep latency score, habitual sleep efficiency score, sleep disturbance score, and use of sleep medication [30]. The variation in sleep problems in the current study and those reported in Iran and Japan may be attributed to the age of the study participants, who were younger in our study.

We also found that there were increased odds of high blood pressure, abdominal obesity, and low HDL cholesterol in participants with poor sleep quality. Poor quality of sleep has been associated with blood pressure in children and adolescents from Brazil [31] and the United States [28]. Poor quality of sleep has been reported to increase BP through increased sympathetic activity and/or reduced parasympathetic activity, which may lead to a sympathovagal imbalance in patients with hypertension [32], myocardial infarction [14], and in children at risk to obesity [13]. The association between poor quality of sleep and anthropometric indices has been reported to be inconsistent in meta-analysis with some studies favouring a positive association, while other studies, especially in college students, reported no association [33]. Consistent with the findings of this study, poor quality of sleep was associated with obesity and/or overweight in college students in the United States and Korea [15, 19], as well as Hispanic students [34]. Contrary to the findings of this study, Vargas et al. (2014) reported that poor quality of sleep was not associated with overweight/obesity status derived from self-reported weight and height among college students in the United States [35]. Similar findings were reported by Vasconcelos et al. (2013) among college students in Brazil [21].

In this study, we found no association between the quality of sleep and the level of physical activity. This is consistent with the findings of Kakinami et al. (2017) who reported no association between physical activity and poor quality of sleep in Canadian university students [8]. A similar finding was reported in an international study involving more than 15,000 university students in 23 countries across Africa, Asia, and the Americas [7]. In contrast to our findings, systematic reviews indicate that many investigators have reported a positive association between levels of physical activity and sleep in young adults [6]. Moreover, longitudinal studies in Swedish adults indicate that sleep complaints are reduced with increased physical activity at baseline and follow-up study as well [36]. These findings suggest that sleep-promoting effects of high physical activity in young adults might be less based on behavioural patterns, but rather depend on individual appraisals about being sufficiently physically active and fit [29]. This finding is in line with cognitive models of insomnia that point to the important role of cognitive processes such as attention, perception, memory, reasoning, belief, attribution, and expectations in the onset and maintenance of sleep complaints [7, 19, 35]. Because the biological plausibility of a sleep-promoting impact is still not sufficiently established, the paradoxical effects of physical activity levels are not a surprise.

The limitations of this study include that it is cross-sectional in design which precludes any inference on causality. Hence, we cannot conclude that poor quality of sleep leads to the development of MetS or vice versa. A longitudinal design may be required to confirm this association. Secondly, the study was conducted in university students in an urban city in Ghana. This approach was convenient for participants' recruitment, as well as administration of questionnaires since the online English form of PQSI and IPAQ-SF were used. However, university students in Ghana have a different socioeconomic profile and environmental stresses compared to the general population, even within the same age group. This affects the generalizability of our findings. Moreover, we did not measure other factors that may affect the quality of sleep and MetS such as anxiety, depression, socioeconomic status, and others. Lastly, quality of sleep and physical activity levels, assessed using a validated questionnaire, may be subjected to recall bias.

## 5. Conclusion

Our study has shown that in young adults in university, there is a high prevalence of poor quality of sleep associated with MetS and obesity. Physical activity status was not associated with poor quality of sleep. Future studies may utilize longitudinal design and use objective methods of assessing the quality of sleep and physical activity levels, to investigate the roles of sleep and physical activity in the development of MetS in young adults from the sub-Sahara Africa population.

## Figures and Tables

**Table 1:** General characteristics of study participants by gender.

	All participants (n =340)	Good sleepers (n =156)	Poor sleepers (n =184)	p
Age, years	23.9 ± 2.8	23.7 ± 2.7	24.1 ± 2.8	0.148
Males, n (%)	120 (35.3)	62 (39.7)	58 (31.5)	0.114
Smoking status, n (%)				0.221
Current	6 (1.8)	2 (1.1)	4 (2.6)	
Former	16 (8.7)	6 (3.3)	10 (6.4)	
Never	318 (93.5)	176 (95.6)	142 (91)	
Alcohol status, n (%)	46 (13.5)	16 (10.3)	30 (16.3)	0.104
Weight, kg	74.8 ± 10.9	73.6 ± 10.9	76.2 ± 11.1	0.031
Height, cm	168 ± 7	168 ± 7	169 ± 7	0.126
BMI, kg/m^2^	26.5 ± 4.1	25.7 ± 4.2	27.3 ± 4	<0.001
Waist circumference, cm	87 ± 20	85 ± 20	88 ± 20	0.169
Hip circumference, cm	105 ± 18	103 ± 19	106 ± 18	0.136
Waist-hip ratio	0.83 ± 0.16	0.8 ± 0.16	0.85 ± 0.17	0.006
Systolic BP, mmHg	119 ± 15	118 ± 15	121 ± 15	0.067
Diastolic BP, mmHg	76 ± 7	75 ± 7	76 ± 9	0.25
Pulse BP, mmHg	43 ± 12	42 ± 12	43 ± 11	0.424
Mean BP, mmHg	90 ± 9	89 ± 9	92 ± 9	0.002
FPG, mmol/l	5 ± 1	4.9 ± 1.2	5.1 ± 0.8	0.068
Triglycerides, mmol/l	0.76 ± 0.29	0.8 ± 0.3	0.7 ± 0.3	0.337
Total cholesterol, mmol/l	5.2 ± 1.1	4.9 ± 1.1	5.5 ± 1	<0.001
HDL cholesterol, mmol/l	1.26 ± 0.15	1.6 ± 0.2	1.1 ± 0.1	<0.001
LDL cholesterol, mmol/l	3.6 ± 1.2	3.5 ± 1.3	3.7 ± 1.1	0.06
Urea, mmol/l	3.4 ± 0.8	3.4 ± 0.8	3.5 ± 0.9	0.284
Creatinine, mmol/l	90.7 ± 16.1	90.8 ± 15.2	90.6 ± 17.1	0.912
MetS, n (%)	42 (12.4)	12 (7.7)	30 (16.2)	0.016
Physical activities, median (IQ)				
Duration, min/week	16 (11 – 50)	18 (10 – 61)	16 (11 – 46)	0.327
Low, METs-min/week	198 (66 – 231)	198 (60 – 231)	181 (66 – 201)	0.813
Moderate, METs-min/week	360 (240 – 360)	372 (231 – 401)	299 (198 – 476)	0.288
Vigorous, METs-min/week	480 (318 – 542)	498 (475 – 502)	468 (460 – 783)	0.47
Total, METS-mins/week	396 (66 – 924)	388 (51 – 918)	429 (73 – 924)	0.742

BMI, body mass index; BP, blood pressure; FPG, fasting plasma glucose; HDL, high density lipoprotein cholesterol; LDL, low density lipoprotein cholesterol; MetS, metabolic syndrome; METs, metabolic equivalent of tasks.

**Table 2:** Comparison of sleep indices among participants with or without metabolic syndrome.

	MetS absent (n =42)	MetS present (n =298)	p
Subjective sleep quality	1.83 ± 1.22	1.4 ± 1.38	0.03
Sleep latency	1.56 ± 0.5	1.6 ± 0.5	0.616
Sleep duration	1.15 ± 0.99	1.19 ± 0.99	0.801
Habitual sleep efficiency	0.89 ± 0.16	1.28 ± 0.34	<0.001
Sleep disturbance	1 ± 0.06	1 ± 0.01	0.999
Sleep medication	1.4 ± 1.38	1.82 ± 1.22	0.034
Daytime dysfunction	0.57 ± 0.49	0.6 ± 0.5	0.707
Global PSQI score	5.32 ± 1.23	8.1 ± 2	<0.001

PSQI, Pittsburgh sleep quality index.

**Table 3:** Prevalence of components of metabolic syndrome by sleep status of participants.

	Good sleepers (n =156)	Poor sleepers (n =184)	OR (95% CI)	p
All participants (n =340)				
Impaired fasting glucose	25 (11.5)	42 (22.8)	1.23 (0.7 – 2.16)	0.471
High blood pressure	46 (29.5)	74 (39.8)	1.61 (1.02 – 2.53)	0.04
Abdominal obesity	19 (12.2)	39 (21.2)	2.08 (1.15 – 3.77)	0.016
Reduced HDL cholesterol	37 (23.7)	78 (42.4)	2.37 (1.48 – 3.79)	<0.001
Hypertriglyceridemia	1 (0.6)	3 (1.6)	2.54 (0.26 – 24.63)	0.08

Males (n =120)				
Impaired fasting glucose	9 (14.5)	11 (19)	1.38 (0.53 – 3.62)	0.514
High blood pressure	16 (25.8)	24 (41.4)	2.03 (0.94 – 4.39)	0.073
Abdominal obesity	6 (9.7)	8 (13.8)	1.49 (0.48 – 4.5)	0.485
Reduced HDL cholesterol	3 (4.8)	9 (15.5)	3.61 (0.93 – 14.08)	0.064

Females (n =220)				
Impaired fasting glucose	16 (17)	26 (20.6)	1.56 (0.75 – 3.23)	0.232
High blood pressure	30 (31.9)	50 (39.7)	1.4 (0.8 – 2.46)	0.234
Abdominal obesity	13 (13.8)	31 (24.6)	2.03 (1.06 – 4.14)	0.041
Reduced HDL cholesterol	34 (36.2)	69 (54.8)	2.14 (1.24 – 3.69)	0.007
Hypertriglyceridemia	1 (1.1)	3 (2.4)	2.27 (0.23 – 22.16)	0.481

HDL, high density lipoprotein cholesterol.

**Table 4:** Association between sleep status versus metabolic syndrome and physical activity level in multivariable logistic regression models.

	Unadjusted OR	P	Adjusted OR	p
All participants				
MetS	2.34 (1.15 – 4.74)	0.016	2.08 (1.09 – 5.17)	0.039
Low PA	1.22 (0.75 – 2)	0.421	1.39 (0.81 – 2.3)	0.238
BMI categories (reference: Normal BMI)				
Overweight	1.03 (0.61 – 1.74)	0.675	0.99 (0.58 – 2.34)	0.862
Obese	1.92 (1.07 – 3.43)	0.028	1.32 (1.02 – 3.56)	0.043
Males				
MetS	2.16 (0.62 – 7.49)	0.225	2.72 (0.76 – 9.76)	0.124
Low PA	1.3 (0.63 – 2.69)	0.481	1.38 (0.66 – 2.89)	0.39
BMI categories (reference: Normal BMI)				
Overweight	0.81 (0.32 – 2.03)	0.651	0.76 (0.25 – 2.78)	0.753
Obese	1.62 (0.79 - 3.32)	0.059	1.42 (0.71 – 3.97)	0.096
Females				
MetS	2.23 (1.11 – 4.45)	0.024	2.11 (1.04 – 4.25)	0.038
Low PA	1.32 (0.63 – 2.74)	0.456	1.36 (0.65 – 2.85)	0.41
BMI categories (reference: Normal BMI)				
Overweight	1.3 (0.67 – 2.53)	0.437	1.18 (0.61 – 3.07)	0.653
Obese	1.62 (0.79 - 3.32)	0.186	1.29 (0.73 – 3.95)	0.214

MetS, metabolic syndrome; PA, physical activity; OR, odds ratio.

## Data Availability

Data supporting the conclusions of this paper is available and can be requested from the lead author (Dr Kwame Yeboah).

## Abbreviations

MetS: Metabolic syndrome
CVDs: Cardiovascular diseases
PSQI: Pittsburgh sleep quality index
IPAQ-SF: International physical activity questionnaire-short form
BMI: Body mass index
BP: Blood pressure
LDL: Low-density lipoprotein
HDL: High-density lipoprotein.
